# Current Opinion on Peritoneal Carcinomatosis Treatment: a Survey of the Indian Society of Peritoneal Surface Malignancies (ISPSM)

**DOI:** 10.1007/s12029-020-00538-1

**Published:** 2020-10-19

**Authors:** David Martin, F. Grass, S. V. S. Deo, K. R. Ashwin, A. Maheshwari, M. Hübner, S. P. Somashekhar

**Affiliations:** 1 Department of Visceral Surgery, Lausanne University Hospital CHUV, Lausanne, Switzerland; 2 Department of Surgical Oncology, All India Institute of Medical Sciences, New Delhi, India; 3 Department of Surgical Oncology, Manipal Hospital, Bangalore, India; 4 Department of Gynecological Oncology, Tata Memorial Cancer Hospital, Mumbai, India

**Keywords:** Peritoneal carcinomatosis, Treatment, Survey

## Abstract

**Purpose:**

Patients with peritoneal carcinomatosis (PC) are increasingly treated with multidisciplinary combined approaches. The study aim was to assess current practice and perceptions of treatment modalities of PC.

**Methods:**

Indian Society of Peritoneal Surface Malignancies (ISPSM) members were invited to complete an online survey. Current practice and perceptions of treatment modalities were assessed through 19 closed questions. Scores were assessed using a Likert scale (0: not important, 5: very important). Treatment modality satisfaction was assessed using a semantic scale (frustrated: 0, perfectly happy: 10). Participants were sent 3 reminders at 4-week intervals.

**Results:**

Fifty-seven out of 182 members completed the survey (31%). Forty percent of participants had an experience of at least 10 years, and 75% stated treating less than 20 PC patients per year. Main treatment goals for patients with PC were cure (5/5) and symptom relief (4/5). Participant’s satisfaction with treatment modalities for ovarian, colorectal, and gastric PC were 6/10, 5/10, and 2/10, respectively. Hyperthermic intraperitoneal chemotherapy (HIPEC) for ovarian (57%) and colorectal (44%) origins were considered to be useful. Clinical usefulness of chemotherapy for gastric PC was rated to be low (17%).

**Conclusions:**

Current treatment modalities fall short to satisfy the needs (cure, symptom relief) of patients with PC. Alternative systemic and intraperitoneal treatment modalities should be assessed.

**Electronic supplementary material:**

The online version of this article (10.1007/s12029-020-00538-1) contains supplementary material, which is available to authorized users.

## Introduction

The prognosis for peritoneal carcinosis (PC) is poor and the response to systemic chemotherapy limited [1]. Intraperitoneal treatment has brought some progress, especially for ovarian cancer [2]. Several studies reporting on systemic chemotherapy and symptom-directed surgery without cytoreduction demonstrated a median survival ranging from 3 to 7 months for patients with PC from non-gynecologic malignancies [3, 4]. The combination of systemic chemotherapy after extensive cytoreductive surgery (CRS) with concurrent heated intraperitoneal chemotherapy (HIPEC) has demonstrated a remarkable improvement in survival of highly selected patients over the last two decades, with even a chance for long-term survival [5–8]. However, these procedures have high morbidity and mortality, and many patients are not eligible. Furthermore, role of HIPEC remains unclear and its effectiveness seems limited due to poor distribution and penetration of chemotherapy [9, 10].

More recently, targeted drugs and immunotherapy but also alternative intraperitoneal options such as pressurized intraperitoneal aerosol chemotherapy (PIPAC) and neoadjuvant intraperitoneal and systemic chemotherapy (NIPS) have become available. PIPAC is a novel minimal-invasive approach for intraperitoneal drug delivery. Administration as an aerosol allows for better distribution within the abdominal cavity, and tissue concentrations of the therapeutic agents are increased [11]. NIPS is a bidirectional chemotherapy regimen that has been developed to reduce the volume and peritoneal cancer index of PC [12]. However, specific evidence-based guidelines and consensus on optimal treatment strategy for PC are lacking and vary widely.

The aim of this study was to assess practice and perceptions regarding PC, as well as satisfaction with available treatment modalities.

## Methods

This is a qualitative study among Indian Society of Peritoneal Surface Malignancies (ISPSM) members. The network is composed of 182 members who are involved in the care of PC. The questions concerned demographics, current practice, and perceptions concerning available treatment modalities for PC. The same survey has previously been distributed among a Swiss oncology network in 2017, and same methodology was used in the present study [13].

Treatment goals were evaluated by the participants, and overall scores were obtained depending on their rating on a Likert scale (0: not important, 5: very important). Satisfaction with treatment modalities was measured using a semantic scale (0: frustrated, 10: perfectly happy). A similar scale was used to assess new treatment needs for PC (0: no need, 10: urgent need). Usefulness of chemotherapy and HIPEC for different origins of PC was assessed with closed questions and 3 possible answers (poor, moderate, and high). Nineteen questions were included in the survey (supplementary material). An online software was used (Survey Monkey®) with email distribution. Three reminders were sent at 4-week intervals.

Numbers and percentages were used for categorial variables, while medians and interquartile ranges (IQR) were used for continuous variables.

## Results

Fifty-seven out of 182 members completed the survey, yielding a response rate of 31%. Demographics are presented in Table 1. Most participants (*n* = 23, 40%) had an experience of at least 10 years and vast majority (*n* = 43, 75%) treated less than 20 PC patients annually. Thirty-four participants (60%) worked in centers offering HIPEC treatments, while 15 in centers offering PIPAC (26%).

**Table 1:** Participant demographics

	Overall, *n* = 57
Speciality	
Medical oncologists	3 (5%)
Gynaecologic surgical oncologists	25 (44%)
Gastrointestinal surgical oncologists	29 (51%)
Years since board qualification *	
< 5 years	22 (39%)
5–10 years	12 (21%)
> 10 years	23 (40%)
Patients with PC personally treated annually	
< 10	29 (51%)
10–20	18 (32%)
20–50	7 (12%)
> 50	3 (5%)
Annual number of HIPEC procedures at institution	
0	23 (40%)
< 10	11 (19%)
10–20	9 (16%)
20–50	10 (18%)
> 50	4 (7%)

*PC* peritoneal carcinomatosis, *HIPEC* hyperthermic intraperitoneal chemotherapy

*Oncology, surgical oncology, or other specialist degree or fellowship

**Fig 1:**
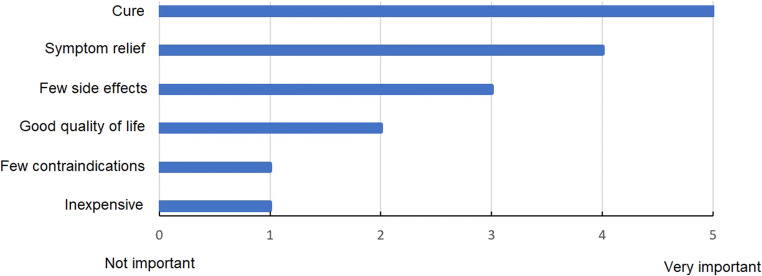
Main goals for the treatment of patients with peritoneal carcinomatosis

**Fig 2:**
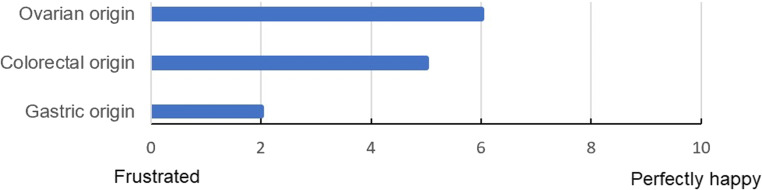
Satisfaction with available treatment options for peritoneal carcinomatosis

Main goals for the treatment of patients with PC were cure and symptom relief (Fig. 1). The need for new treatment approaches was high (8/10, IQR 7–10). Participants’ satisfaction with treatment modalities for different PC origins is displayed in Fig. 2.

Usefulness of chemotherapy and HIPEC as treatments for PC of different origins is shown in Table 2. HIPEC for PC of ovarian (high; 57%) and colorectal (high; 44%) origins were considered useful, while systemic chemotherapy for gastric PC was considered not very useful (poor; 57%).

**Table 2:** Clinical usefulness of chemotherapy and HIPEC

	Poor	Moderate	High
Systemic chemotherapy			
Ovarian origin, *n* (%)			
As second line treatment	5 (7%)	26 (46%)	26 (46%)
As third line treatment	20 (35%)	20 (35%)	17 (30%)
Colorectal origin, *n* (%)			
As first line treatment	14 (24%)	32 (57%)	11 (19%)
As second line treatment	15 (26%)	34 (60%)	8 (13%)
Gastric origin, *n* (%)			
As first line treatment	32 (57%)	15 (26%)	10 (17%)
As second line treatment	38 (67%)	14 (24%)	5 (9%)
HIPEC			
Ovarian origin	5 (7%)	20 (36%)	32 (57%)
Colorectal origin	7 (12%)	25 (44%)	25 (44%)
Gastric origin	23 (40%)	26 (45%)	8 (15%)

*HIPEC* hyperthermic intraperitoneal chemotherapy, *PC* peritoneal carcinomatosis

## Discussion

This study identified cure and symptom relief as main treatment goals for patients with PC. Participant’s satisfaction with available treatment options for colorectal and gastric PC was low, and most participants expressed a need for new treatment modalities. HIPEC and systemic chemotherapy were considered most useful options for ovarian and colorectal PC, while their utility in gastric PC seemed less convincing.

The ISPSM network is a national group with quite variable experience in the treatment of PC considering patient accrual, as illustrated by the demographics of this current study. The participants were mainly surgical oncologists (95%) and majority (75%) treated less than 20 PC patients per year. A recent retrospective study on CRS/HIPEC procedures showed that postoperative morbidity could be improved through centralization in high volume centers, with a threshold of 45 cases per year [14]. Another multicentric study demonstrated that centers with more than 7 years of experience in the treatment of PC had lower postoperative morbidity and better overall survival [15]. However, patient’s registration and surgery characteristics are specific to each country, which limits comparisons and the creation of homogeneous registers [16]. Otherwise, the vision of PC and its various treatments seems to differ between oncologic surgeons and medical oncologists [17]. Indications are controversial, which is reflected by differences in national guidelines and in the numbers of treated patients between individual hospitals, regions and countries [17]. Thus, it is essential to discuss treatment strategies in the setting of multidisciplinary team meetings as done in the vast majority of expert centers [18].

The concepts of centralization in order to increase the intermediate and high volume centers (> 30 cases/year), training the surgeons with less experience in courses and the fact of addressing the most complex procedures to expert centers have been shown to be beneficial in reducing postoperative morbidity and increasing survival in various type of cancers [19, 20]. Unfortunately, this study did not assess how participants felt about their specific training for the treatment of PC.

In the present study, main treatment goals of PC were cure and symptom relief, which is comparable to a former survey among Swiss oncologists [13]. A retrospective study reported that improved survival and preserving quality of life by reducing both disease-related symptoms and therapy-related side-effects were important treatment goals [21]. These goals were considered moderately important in this survey, which furthermore showed that the economic aspect was not important at all. A previous study on quality of life after PIPAC showed that other requirements for optimal PC treatment included oncological efficacy (tumor response, survival) but also low toxicity and few side effects [22]. These observations may reflect a gap between physicians' expectations and the history of the disease, with cost effectiveness being questioned [13].

Usefulness of chemotherapy and HIPEC was heterogeneous in this survey. However, HIPEC and systemic chemotherapy were considered most useful for ovarian and colorectal PC, while their use in gastric PC was questioned. A recent worldwide web-based survey conducted amongst experts in 19 countries estimated that currently more than 3800 patients with PC (synchronous and metachronous) were annually treated with CRS and HIPEC in 430 centers [18]. In a randomized trial, CRS followed by HIPEC improved survival in patients with PC of colorectal origin if complete cytoreduction could be performed [23]. Eight-year follow-up of this cohort confirmed the significant increase of survival time, even with a possibility of long-term survival [6]. Another randomized study showed that CRS with intraperitoneal chemotherapy may be superior to systemic oxaliplatin-based treatment of colorectal cancer with resectable isolated peritoneal metastases [24]. Furthermore, the therapeutic approach combining CRS with perioperative intraperitoneal chemotherapy can be performed with acceptable morbidity and mortality [25]. Definite curative potential of approximately 28% has been reported in a colorectal PC cohort including 67 patients [26]. In an international survey, experts currently considered CRS and HIPEC to be a treatment with curative intent in colorectal PC [18]. In this survey, the role of CRS and HIPEC in gastric cancer was found to be questionable, although the evidences have suggested their role in patients with a low peritoneal disease burden that can be completely reduced and in particular those of diffuse-mixed type, with serosal invasion [27, 28].

For PC of ovarian origin, a systematic review showed that intraperitoneal chemotherapy increases overall survival and progression-free survival [29]. More recently, a randomized study showed that the addition of HIPEC to interval CRS resulted in longer recurrence-free survival and overall survival than surgery alone and did not result in higher rates of side effects [30]. Concerning the treatment of PC of gastric origin, the therapeutic approach combining CRS and intraperitoneal chemotherapy may achieve long-term survival, but the high mortality rate (6.5%) underlines the need for strict selection criteria (limited and resectable PC) and exclusive treatment by experienced institutions [31].

The obvious medical need for new and better therapeutic options for PC was strongly expressed in this study. A systemic review including 21 national and international guidelines showed that the treatment strategy for PC of colorectal origin was not extensively described and evidence was often insufficient [32]. Thus, national guidelines vary, resulting in large treatment disparities between countries [18]. PIPAC is a minimally invasive approach representing a novel treatment for patients with PC of various origins, and preclinical data suggested better distribution and higher tissue concentrations of chemotherapy agents compared with conventional intraperitoneal chemotherapy by lavage [11, 33]. Yet currently, PIPAC represents an alternative for patients with advanced PC and not eligible for radical treatment [18]. Another novel multidisciplinary treatment combining neoadjuvant bidirectional intraperitoneal/systemic chemotherapy (NIPS) has been recently developed [34–36]. Complete cytoreduction is often difficult when the peritoneal cancer index (PCI) score is high or there is extensive involvement of the small bowel mesentery. Thus, NIPS is proposed for PCI reduction, eradication of free peritoneal floating cancer cells, and pathological response before CRS [36]. New modalities might also include intraperitoneal immunotherapy, which is particularly interesting due to the wide range of immune competence of the peritoneal cavity [13, 37]. The wide variety of anticancer immunotherapeutic strategies are now garnering attention for control of regional disease of the peritoneal cavity [38].

This has several limitations that need to be addressed. The ISPSM network is a national group mainly composed of surgical oncologists which might lead to an overrepresentation of surgical treatment choices. The specialties of members who did not answer the survey are unknown, as are the reasons why they did not respond. The response rate was rather low despite 3 reminders, leading to possible selection bias. However, the response rate to this survey was higher (31%) than other previous surveys performed targeting similar networks (23-28%) [17, 39]. Concerning healthcare system, a large proportion of Indian centers do not have access or cannot afford all treatment modalities, which may have introduced selection bias. Indeed, ISPSM members who responded to the survey had varied experiences, with limited access to treatment (for example, 40% do not have the possibility of carrying out HIPEC procedures in their center); thus, this constitutes a certain heterogeneity among the cohort and limits interpretation and generalization. It should also be noted that the various specialists had access to all questions, even those outside their specialty, which potentially introduced systematic errors. Finally, due to their rarity, primary peritoneal cancers (pseudomyxoma peritonei and peritoneal mesothelioma) have not been addressed.

In conclusion, main treatment goals of PC were cure and symptom relief. Furthermore, this survey pointed out a lack of satisfaction with treatment approaches and alternative systemic and intraperitoneal modalities should be assessed**.**

## Electronic supplementary material

ESM 1 (DOCX 113 kb).
