# Supplementary material for: Current Opinion on Peritoneal Carcinomatosis Treatment: a Survey of the Indian Society of Peritoneal Surface Malignancies (ISPSM)
Source: J Gastrointest Cancer. 2020 Oct 19;52(3):1061–6. doi: 10.1007/s12029-020-00538-1 (PMC8376720; doi:10.1007/s12029-020-00538-1)
Supplement: Supplementary file 1 — (DOCX 113 kb). [file 12029_2020_538_MOESM1_ESM.docx]

**Current opinion on peritoneal carcinomatosis treatment: a survey of the Indian Society of Peritoneal Surface Malignancies (ISPSM)**

**1. What is your medical profession? (several answers are possible)**

Gastrointestinal Surgeon

Gynecologic Surgeon

Medical Oncologist

Other (please specify):

**2. How much time are you in practice since board qualification?**

< 5 years

5-10 years

> 10 years

**3. How many patients with peritoneal carcinomatosis do you personally treat per year?**< 20

20-50

50-100

> 100

**4. What is the annual number of hyperthermic intraperitoneal chemotherapy (HIPEC) procedures at your institution? w**

0

< 10

10-20

20-50

> 50

**5. Does your department offer the following treatment options for peritoneal carcinomatosis:**

Hyperthermic intraperitoneal chemotherapy (HIPEC)

Intraperitoneal catheter therapy

Pressurized intraperitoneal aerosol chemotherapy (PIPAC)

Low dose radiotherapy

None

***The next few questions concern the systemic treatment of peritoneal carcinomatosis of various origins.
Please feel free to answer all questions regardless of your medical speciality.***

**6. What are your main goals/priorities for the treatment of your patients with peritoneal carcinomatosis?

Please rate importance of following statements on a Likert scale from 0 (not important) to 5 (very important).**

Cure

Symptom relief

Few side effects

Few contraindications

Inexpensive

Good quality of life

**7. How would you describe the clinical usefulness of systemic chemotherapy in the treatment of resectable peritoneal carcinomatosis of colorectal origin?

- As first line treatment**

Poor

Moderate

High

**8. How would you describe the clinical usefulness of systemic chemotherapy in the treatment of resectable peritoneal carcinomatosis of colorectal origin?

- As second line treatment**

Poor

Moderate

High

**9. How would you describe the clinical usefulness of systemic chemotherapy in the treatment of resectable peritoneal carcinomatosis of gastric origin?

- As first line treatment**

Poor

Moderate

High

**10. How would you describe the clinical usefulness of systemic chemotherapy in the treatment of resectable peritoneal carcinomatosis of gastric origin?

- As second line treatment**

Poor

Moderate

High

**11. How would you describe the clinical usefulness of systemic chemotherapy in the treatment of resectable peritoneal carcinomatosis of ovarian origin?

- As second line treatment**

Poor

Moderate

High

***The next few questions concern the surgical treatment of peritoneal carcinomatosis with Cytoreductive Surgery and Hyperthermic Intraperitoneal Chemotherapy (HIPEC).
Please feel free to answer all questions regardless of your medical speciality.***

**12. How would you describe the clinical usefulness of systemic chemotherapy in the treatment of resectable peritoneal carcinomatosis of ovarian origin?

- As third line treatment**

Poor

Moderate

High

**13. How would you describe the clinical usefulness of HIPEC in the treatment of isolated resectable peritoneal carcinomatosis of ovarian origin?**

Poor

Moderate

High

**14. How would you describe the clinical usefulness of HIPEC in the treatment of isolated peritoneal carcinomatosis of colorectal origin?**

Poor

Moderate

High

**15. How would you describe the clinical usefulness of HIPEC in the treatment of isolated peritoneal carcinomatosis of gastric origin?**

Poor

Moderate

High

***The next questions try to evaluate your satisfaction with available treatment options for peritoneal carcinomatosis.
Please feel free to answer all questions regardless of your medical speciality***

*** 16. Please indicate on a Visual Analogue Scale the need (0=no need, 10=urgent need) for new treatment options for peritoneal carcinomatosis:**

|  | 0 | 1 | 2 | 3 | 4 | 5 | 6 | 7 | 8 | 9 | 10 |
| --- | --- | --- | --- | --- | --- | --- | --- | --- | --- | --- | --- |
|  |  |  |  |  |  |  |  |  |  |  |  |

**17. Please indicate on a Visual Analogue Scale from 0 (=frustrated) to 10 (=perfectly happy)  your satisfaction with available treatment options for peritoneal carcinomatosis of ovarian origin:**

|  | 0 | 1 | 2 | 3 | 4 | 5 | 6 | 7 | 8 | 9 | 10 |
| --- | --- | --- | --- | --- | --- | --- | --- | --- | --- | --- | --- |
|  |  |  |  |  |  |  |  |  |  |  |  |

**18. Please indicate on a Visual Analogue Scale from 0 (=frustrated) to 10 (=perfectly happy)  your satisfaction with available treatment options for peritoneal carcinomatosis of colorectal origin:**

|  | 0 | 1 | 2 | 3 | 4 | 5 | 6 | 7 | 8 | 9 | 10 |
| --- | --- | --- | --- | --- | --- | --- | --- | --- | --- | --- | --- |
|  |  |  |  |  |  |  |  |  |  |  |  |

**19. Please indicate on a Visual Analogue Scale from 0 (=frustrated) to 10 (=perfectly happy)  your satisfaction with available treatment options for peritoneal carcinomatosis of gastric origin:**

|  | 0 | 1 | 2 | 3 | 4 | 5 | 6 | 7 | 8 | 9 | 10 |
| --- | --- | --- | --- | --- | --- | --- | --- | --- | --- | --- | --- |
|  |  |  |  |  |  |  |  |  |  |  |  |
